# 

**Published:** 1903-10-17

**Authors:** 


JUST ISSUED. EIGHTH EDITION. Crown 8vo, 10s. 6d.

### PHARMACY, MATERIA MEDICA AND THERAPEUTICS

Is now rewritten and printed in large type, and is made a companion to the following volume. It contains an account of the action **of every drug in the B.P.**, as well as a section upon all the newest drugs, with an Introduction to the Art of Dispensing. With woodcuts, prescriptions, &c.

Also FOURTH EDITION. 1055 pages, 8vo. 16s.

### DICTIONARY OF TREATMENT.

Containing a Revised and up-to-date Article upon every Diseased Condition, whether Medical, Surgical, Gynæcological, or Ophthalmological, as well as Articles upon Poisoning, Drowning, and the various Surgical Emergencies and Accidents, arranged for Rapid Reference.

By W. WHITLA, M.A., M.D., Professor of Materia Medica, Queen's College, Belfast; Senior Physician to the Royal Victoria Hospital, &c.

The Publisher will supply both volumes, carriage paid, for 21/-

London: HENRY RENSHAW, 356 Strand.

WORKS BY Mr. McADAM ECCLES, M.S., F.R.C.S.,

Assistant Surgeon St. Bartholomew's Hospital, &c.

### HERNIA.

Second Edition, 1902. Price 7/6 net. Illustrated by Numerous Photographs.

### THE IMPERFECTLY DESCENDED TESTIS.

Just published. Profusely Illustrated. Price 7/6.

London: BAILLIÈRE, TINDALL & COX.

### WORKS BY DAVID WALSH, M.D.

RONTGEN RAYS IN MEDICAL WORK. *Third Edition.* 12s. 6d. net. Baillière & Cox, London.

THE HAIR AND ITS DISEASES. 2s. 6d. net. Baillière & Cox.

GOLDEN RULES OF SKIN PRACTICE. 1s. net. Wright & Co., Bristol.

AGE AND OLD AGE: a Handbook. 2s. 6d. R. A. Everett & Co., London.

**THE HANDBOOK OF THE OPEN-AIR TREATMENT**

By Dr. Charles Reinhardt

(Visiting Physician to Hailey Sanatorium, Wallingford, and Stourfield Park Sanatorium, Bournemouth),

Contains Views, Descriptions, and Information respecting 30 Sanatoria in Great Britain.

Price: Cloth, 2/6; Paper, 1/- (postage 4d.)

The "HEALTH RESORT" Publishing Office, 379 Strand, London, W.C.

### NOTICE TO CORRESPONDENTS.

All MSS., Letters, Books for Review, and other matters intended for the Editor should be addressed The Editor, "The Hospital," The Hospital Buildings, 28 & 29 Southampton Street, Strand, W.C.

The Editor cannot undertake to return rejected MS., even when accompanied by stamped directed envelope.

### Notice to Advertisers and Subscribers.

All Advertisements, Orders for Copies of The Hospital, and Business Communications should be addressed to **THE MANAGER (Not to the Editor),** The Hospital, 28 & 29 Southampton Street, Strand, London, W.C.

The Cost of Subscription, post free, is as follows:—Per week, 3½d.; per quarter, 3s. 9d.; per half-year, 7s. 6d.; per annum, 15s. Foreign and Colonial:—Per annum, 19s. 6d., payable, in all cases, in advance.

**NOTICE.—Vol. XXXIV. of "The Hospital" (April 1903 to September 1903), in handsome cloth binding, will shortly be on sale at the office of this Journal, price 7s. 6d. Cases for binding the Numbers contained in this Volume 1s. 6d. Index to Vol. XXXIV., 2½d., post free.**

**The Monthly part for October is now ready. Price 1s., by post, 1s. 3d.**

### The Student of Medicine.

### APPOINTMENTS.

**E. E. Argles**, M.R.C.S., L.R.C.P.Lond., Second Assistant Medical Officer to the St. Marylebone Infirmary. **Thomas Ballantyne**, M.B., Ch.B.Glasg., Junior House Surgeon of the Blackburn and East Lancashire Infirmary. **C. Braine-Hartnell**, M.R.C.S., L.R.C.P., Clinical Assistant to the Chelsea Hospital for Women. **Walter Briggs**, M.B., Ch.B.Vict., Senior House Surgeon of the Blackburn and East Lancashire Infirmary. **R. C. Brown**, M.B., Honorary Anæsthetist to the Alfred Hospital, Melbourne. **J. Walter Browne**, M.B., B.Ch.Dub., Port Health Officer for the Port of Hookianga, New Zealand. **J. C. Curtis**, L.R.C.P., L.R.C.S., Clinical Assistant to the Chelsea Hospital for Women. **D. H. Darbyshire**, M.R.C.S., L.R.C.P., Health Officer at Buckland Hill, West Australia. **A. Duncan**, L.R.C.P.&S.Edin., Certifying Factory Surgeon for the Moorfields District, co. Antrim. **J. T. Edwards**, M.R.C.S.Eng., L.R.C.P.Lond., House Surgeon to the Aberystwyth Infirmary and Cardiganshire General Hospital. **Ignatius J. Flynn**, M.D., M.S.R.U.I., Health Officer at Bunbury, West Australia. **Richard R. Harvey**, M.B., Ch.B.Melb., Health Officer at Norseman, West Australia. **Claud D. Henry**, M.B., B.S.Camb., Police Surgeon at Wellington, New Zealand. **T. H. Hewitt**, L.R.C.P.&S.I., District Medical Officer of the Bridgnorth Union. **Wilfred Knight**, M.B., L.R.C.P.E., has been appointed a Resident Medical Officer to the Durban Hospital, Natal. **R. F. Llewellyn**, M.B., M.Ch.Syd., Government Medical Officer and Vaccinator at Braidwood, New South Wales. **T. Frederick Pearse**, M.D., F.R.C.S.Eng., M.R.C.P.Lond., D.P.H.Camb., Lecturer on Hygiene at the Medical College, Calcutta. **J. A. Rougham**, M.B., B.S.R.U.I., Health Officer at Narrogin, West Australia. **H. Shelmerdine**, M.B., C.M.Edin., District Medical Officer of the Petersfield Union. **C. V. Smith**, L.S.A., District Medical Officer of the Gainsborough Union. **S. R. Smyth**, L.R.C.P.&S.I., District Medical Officer and Public Vaccinator of Moora, West Australia. **B. J. Ward**, M.R.C.S., L.R.C.P.Lond., First Assistant Medical Officer to the St. Marylebone Infirmary. **James Wood**, M.R.C.S.Eng., L.R.C.P.Lond., L.D.S.Eng., Hon. Dental Assistant Surgeon to the Bolton Infirmary and Dispensary.

### "The Hospital" Institutional Library.

"Hospitals and Asylums of the World." 4 Vols., with a Portfolio of Plans, complete £12 12s.

"Burdett's Hospitals and Charities, 1903." 5s. net.

"Burdett's Official Nursing Directory, 1903." 3s. net.

"Cottage Hospitals, General, Fever, and Convalescent." 10s. 6d.

"Uniform System of Accounts for Hospitals and Public Institutions." New Edition. 4s. net.

"Hospital Expenditure: The Commissariat." 2s. 6d.

"A Legal Handbook for the Use of Hospital Authorities." 2s. 6d.

"The Cottage Hospital Case Book or Register of Patients." 10s. and 12s. 6d.

All these are published by the Scientific Press, Ltd., and may be obtained through any bookseller, or direct from the publishers, 28 and 29 Southampton Street, Strand, London, W.C.

### Medical Appointments.

MEDICAL DEPARTMENT OF THE NAVY.

Admiralty, S.W., September 17th, 1903.

An EXAMINATION of CANDIDATES for entry into the Medical Department of the Royal Navy will be held on the 16th NOVEMBER next and following days at Examination Hall, Thames Embankment.

The forms to be filled up by candidates will be supplied on application to this Department.

H. F. NORBURY, Director General.

(63)

DEVON AND CORNWALL HOMŒOPATHIC HOSPITAL.

The POST of R.M.O. falls vacant December 12th, which offers special advantages to a qualified young man desiring to become acquainted with the principles and practice of homœopathy, or to read for higher exams.

Salary £80 per annum, all found.

Early applications to, and all particulars of, the

HON. SEC.,

15 Lockyer Street,

Plymouth.

### THE NURSING PROFESSION: How and Where to Train.

**By Sir HENRY BURDETT, K.C.B.**

A reliable and up-to-date Guide to Training for the calling of a Nurse. In its pages would-be Nurses will find all the information they require.

New Edition. Price **2/-**, post free **2/4.**

Of all Booksellers or of **THE SCIENTIFIC PRESS, LTD.,** 28 & 29 Southampton Street, Strand, London, W.C.

### For District Nurses & Midwives.

Price post free.

**1/-** — **PRACTICAL HINTS ON DISTRICT NURSING.**
By Amy Hughes,
Superintendent of County Nursing Associations Queen Victoria's Jubilee Institute for Nurses for the Sick Poor.

**2d.** — **THE ADVANTAGES AND PRIVILEGES OF A TRAINED DISTRICT NURSE, AND THE DUTIES OF THE DISTRICT TOWARDS HER.**
An interesting little pamphlet by Sir Henry Burdett, K.C.B.

**Cloth, 1/-; Leather, 1/7** — **THE POCKET CASE-BOOK FOR DISTRICT AND PRIVATE NURSES.**
Suitable for the pocket.

**6d.** — **ON PREPARATION FOR OPERATION IN PRIVATE HOUSES.**
By a Surgeon.

**6/3** — **A PRACTICAL HANDBOOK OF MIDWIFERY.**
By Francis Haultain, M.D.
An invaluable complete and advanced handbook for Practising Midwives.
Bound in leather.

**1/-** — **THE MIDWIVES' POCKET BOOK (with chapter on the Midwives Act).**
A valuable little book by Honnor Morten.

**1/6** — **THE ART OF FEEDING THE INVALID.**
By a Medical Practitioner and a Lady Professor of Cookery.

**1/-** — **THE PHYSIOLOGICAL FEEDING OF INFANTS.**
By Eric Pritchard, M.D.
A scientific Guide to the artificial feeding of infants from birth.

*These books are obtainable of all Booksellers and are published by*
THE SCIENTIFIC PRESS, LTD., 28 and 29 Southampton Street, Strand, London, W.C.,
*who will send their latest Catalogue of Nursing Manuals, post free, to any Nurse on application.*

### WORKS FOR NURSES.

**LESSONS ON MASSAGE.** By Mrs. MARGARET D. PALMER, Manager of the Massage Department and Instructor of Massage to the Nursing Staff of the London Hospital. Second Edition enlarged and revised; pp. xvi. + 262 with 118 Illustrations, plain and coloured. *Just Published.* Price 7/6 net.

**A MANUAL FOR STUDENTS OF MASSAGE.** By Miss M. A. ELLISON, L.O.S. With 50 original Illustrations. Price 3/6 net.

**A MANUAL FOR HOME NURSING.** By BERNARD MYERS, M.D., M.R.C.S., Surgeon and Lecturer to St. John's Ambulance Association. *Just Published.* Price 2/6 net.

**NOTES ON HOME NURSING.** By Miss D. M. GOLDIE, Lecturer on Nursing, Hygiene, &c., to the County Councils of London, Surrey and Middlesex. *Just Published.* Price 1/6 net.

**THE FEEDING AND HYGIENE OF INFANTS AND CHILDREN.** By JOHN McCAW, M.D., M.R.C.P., Physician to the Belfast Hospital for Children. *Just Published.* Price 2/6.

**DISEASES OF THE HAIR, INCLUDING GREYNESS, BALDNESS, &c., AND THEIR TREATMENT.** By DAVID WALSH, M.D., Western Hospital for Diseases of the Skin. Price 2/6 net.

**MENSTRUATION AND ITS DISORDERS.** By ARTHUR E. GILES M.D., B.Sc., F.R.C.S., Surgeon Chelsea Hospital for Women. Price 2/6 net.

**MOVABLE ATLAS OF THE ANATOMY AND PHYSIOLOGY OF THE FEMALE GENERATIVE ORGANS AND OF PREGNANCY.** Second Edition. With Text. By ARTHUR E. GILES, M.D., F.R.C.S. Price 3/- net.

**MOVABLE ATLAS OF THE ANATOMY AND PHYSIOLOGY OF THE CHILD.** With Explanatory Text. By D'ARCY POWER, M.A.Oxon, M.B., F.R.C.S. Price 3/- net.

**BAILLIÈRE'S POPULAR MOVABLE ATLAS OF THE ANATOMY AND PHYSIOLOGY OF MAN.** Size 16 by 7½ inches. With Explanatory Text. By W. S. FURNEAUX, Special Science Teacher London School Board. Price 3/6 net.

London: BAILLIÈRE, TINDALL & COX, 8 Henrietta Street, Covent Garden.

### NOW READY,

A REPRINT OF

### A NURSING HANDBOOK

TOGETHER WITH S. MAW, SON AND SONS'

### COMPLETE CATALOGUE OF NURSING REQUISITES.

The above work is now republished at a cost of 1s. per copy. Messrs. S. M., S. and S. have been compelled to make this charge on account of the extreme popularity of the work and the great expense incurred in its production.

***Post Free on receipt of 1s.***

Nurses' Bags.

### S. MAW, SON and SONS,

7 to 12 ALDERSGATE STREET, LONDON, E.C.

